# Emphasis on the Role of PF4 in the Incidence, Pathophysiology and Treatment of Heparin Induced Thrombocytopenia

**DOI:** 10.1186/1477-9560-11-7

**Published:** 2013-04-05

**Authors:** M Margaret Prechel, Jeanine M Walenga

**Affiliations:** 1 Departments of Pathology and Thoracic & Cardiovascular Surgery, Loyola University Medical Center, Bldg 110, Rm 5225, 2160 S. First Avenue, Maywood, IL 60153, USA

**Keywords:** Heparin Induced Thrombocytopenia (HIT), Platelet Factor 4 (PF4), Heparin

## Abstract

Heparin Induced Thrombocytopenia (HIT) is caused by antibodies that recognize platelet factor 4 (PF4) associated with polyanionic glycosaminoglycan drugs or displayed on vascular cell membranes. These antibodies are elicited by multimolecular complexes that can occur when heparin is administered in clinical settings associated with abundant PF4. Heparin binding alters native PF4 and elicits immune recognition and response. While the presence of heparin is integral to immunogenesis, the HIT antibody binding site is within PF4. Thus HIT antibodies develop and function to cause thrombocytopenia and/or thrombosis only in the presence of PF4. Future emphasis on understanding the biology, turnover and regulation of PF4 may lead to insights into the prevention and treatment of HIT.

## Review

### Introduction

With the introduction of heparin into clinical practice in the 1940s, surgeons were able to perform complex operations using this anticoagulant to prevent and/or treat obstructive clots. The availability, over-all safety and performance of heparin opened the door for procedures such as hemodialysis, open-heart surgery and organ transplants [1]. Today, heparins are used for prophylaxis and treatment of an expanding list of medical conditions and surgical and interventional procedures [2]. An estimated 12 million patients receive some form of heparin each year in the United States [3].

In the decades following its introduction however, a paradoxical, adverse effect of heparin was recognized [4]. In a small percentage of patients, there was an unexplained drop in platelet count after several days of heparin therapy. Typically, low platelet count in the face of anticoagulation poses the risk of a bleeding complication. Instead patients with this “heparin-induced thrombocytopenia” (HIT) were at risk for venous and arterial thrombosis [5]. HIT thrombosis (HITT) necessitates the withdrawal of heparin which would exacerbate rather than resolve this unusual clotting condition. In the absence of an effective alternative therapy, HITT can progress to thromboembolic complications including deep vein thrombosis, pulmonary embolism, myocardial infarction and stroke [6]. Depending on the patient population, HIT occurs in 0.5-5% of patients receiving heparin for 5 or more days. Of patients with HIT, 30-72% develop thrombotic complications with 10% risk of limb amputation and 20-30% risk of death [7]. The difficulty of management and the devastating consequences of the HIT(T) syndrome have encouraged an abundance of research into HIT pathogenesis, with the goal of minimizing the risk for developing HIT(T) and discovering safe and effective alternative anticoagulant drugs [8].

Early investigators determined that the agent responsible for HIT was a platelet-activating antibody that caused platelet consumption and a hypercoagulable state [9]. Initial suspicion of an immune involvement in HIT, based on the 5–15 day interval between heparin exposure and onset of symptoms, was confirmed by demonstrating that HIT patient sera or its IgG fraction caused activation of donor platelets in the presence of heparin in vitro [10,11]. However the “heparin antibody” could not be isolated [12]. After a decade, investigators discovered that the HIT antigen was not heparin itself, but rather a specific complex of heparin with an endogenous platelet protein, platelet factor 4 (PF4) [13-15]. Much research has been devoted to evaluating the specifics of titer, isotype and avidity of PF4/heparin antibodies [16-19] and the characteristics, duration or dosage of heparin-like drug [20-23] that are most closely related to HIT pathology. Far fewer reports deal with the role of PF4 in the risk of immunogenesis (development) and pathogenesis (function) of HIT antibodies. This review emphasizes the central role that the availability of the PF4 antigen plays in HIT.

### Platelet Factor 4

Platelet factor 4 (PF4), also known as chemokine CXCL4, is a cationic, 7.8 kDa protein which forms tetramers at physiological pH and ionic strength [24-26]. PF4 is released from the alpha-granules of activated platelets as a complex with a chondroitin sulfate proteoglyan carrier [27,28]. It disappears rapidly from plasma as it transfers to higher affinity heparan sulfate [29-31] on endothelial cells [32,33], inhibiting local antithrombin (AT) activity and thus promoting coagulation [34]. In addition to its role in hemostasis, PF4 has many other biological effects, which may also depend on its association with extracellular glycosaminoglycans (GAGs); these have been reviewed elsewhere [34-36].

### Role of PF4 in HIT immunogenesis (antibody development)

In addition to the presence of heparin during anticoagulant therapy the formation of immunogenic complexes that provoke antibody depends on the availability of PF4 [37]. The plasma level of PF4 is proportional to the extent and duration of platelet activation and of PF4 turnover, depending to a large extent on the underlying clinical status of each patient [38]. Increased PF4 levels are observed in inflammatory or infectious disease [39,40], diabetes [41], cardiovascular and renal disease [42-44], atherosclerosis [45] and other conditions affecting vascular health [41,46-48] or in response to traumatic medical procedures [49-51] or cardiopulmonary bypass [52]. Upon release from activated platelets, PF4 rapidly associates with heparan sulfate on endothelial cells and can be brought back into circulation by heparin, for which it has a higher affinity [30,31,53]. This heparin-releasable PF4 pool (HR-PF4) can be evaluated by measuring plasma PF4 before and after injection of heparin; subsequent heparin doses release less PF4 for an interval related to the rate that PF4 accumulates on the endothelium [54,55]. HR-PF4 is another measure of PF4 availability. Compared to healthy control subjects, a higher level and rate of re-establishment of the extracellular PF4 has been demonstrated in several patient populations, including those with diabetes [56,57], atherosclerosis [58] renal, [44] cardiovascular or coronary artery disease [59-61]. Underlying disease, especially when associated with platelet activation, impacts the availability of PF4 and the likelihood of formation of multimolecular PF4/heparin complexes.

The availability of PF4 is influenced both by acute and chronic platelet activation, and logically plays a role in the risk for generation of PF4/heparin antibodies in the context of anticoagulant therapy. This suggests an explanation for the common observation that specific patient populations are known to be at an increased risk of developing HIT antibodies [51,62-64]. Thus it is important to recognize that in addition to the type, dose and duration of heparin therapy, there are patient related variables that are important in assessing the risk for generation of HIT antibodies [8,65,66].

### Role of PF4 in HIT pathogenesis (antibody function)

It is well documented in the literature that the presence of HIT antibody does not cause thrombocytopenia or thrombosis in the majority of seropositive patients [62,67-69]. It is when certain HIT antibodies bind their PF4 antigen, forming immune complexes, that subsequent Fc-gamma receptor-mediated platelet activation ensues and can lead to thrombocytopenia and/or thrombosis. Thus the HIT syndrome depends not only on the presence of HIT antibodies of sufficient titer and specificity but also on the presence of the antigenic PF4 target [37]. Many of the conditions that increase the risk of antibody formation by causing platelet activation and release of PF4 (as described above) similarly increase the risk of clinical consequences due to HIT antibody immune complex-mediated platelet activation [51,70].

In contrast to immunogenesis (formation of antibodies), which is dependent on the presence of heparin, HIT pathogenesis (antibody function) can occur after cessation of anticoagulant therapy, referred to as “delayed HIT” [71-76]. Studies have demonstrated that PF4 bound to glycosaminoglycans on the surface of endothelial cells, monocytes and platelets can present the HIT antibody target antigen [65,77-80]. Thus the HIT antigenic target may be available in the absence of heparin, when PF4 from activated platelets associates with GAGs on vascular cells [79]. There is no evidence to suggest that extracellular GAG-associated PF4 initiates antibody formation; however, HIT antibodies resulting from heparin exposure may bind to these sites and form HIT antigen-antibody immune complexes. Indeed, HIT related thrombotic complications often occur at sites of vascular damage from catheter placement or at surgical sites [81-86], where PF4 can accumulate at high levels [29]. Many situations, both during and subsequent to hospitalization, may impact the level of platelet activation and lead to an increase in GAG-associated PF4, and potential HIT target antigen. Chronic health conditions such as atherosclerosis, diabetes or hypercholesterolemia [41,48,75], as well as spontaneous/isolated instances of infection or injury, for example, could change the likelihood of HIT occurring in seropositive patients by increasing platelet activation and the availability of PF4 target antigen [87]. Currently scoring systems, based on evaluation of clinical presentation [88-90], along with laboratory measurements of the titer, isotype and in vitro functionality of PF4/heparin antibodies are the basis of assessing the likelihood of clinical HIT [16,17]. Patient-related factors, including status of platelet activation and PF4 turnover certainly play a role in HIT risk. Further research will be needed to understand how to evaluate these factors to improve risk prediction.

### PF4/Heparin complexes

The development and functionality of HIT antibodies are dependent not only on availability of PF4, but more importantly on the level of PF4 in relation to heparin (or other GAG) [63,91]. The binding of the cationic PF4 tetramer and heparin, or other polymeric anion, occurs by relatively non-specific electrostatic interactions [20,92-94], and the size and characteristics of the resulting complexes are governed by the concentration of each [63]. Numerous in vitro studies have been conducted using antibodies isolated from HIT patients to define the characteristics of PF4/heparin complexes that are most antigenic (cross-reactive). These studies indicate that complexes formed at near equimolar ratios of PF4 and heparin, correlate with optimal antibody binding. With higher proportions of heparin, complexes are smaller and do not bind to form platelet activating HIT antibody immune complexes [63,95-98]. Platelet factor 4 to heparin ratios (PHRs) in the range of 3:1 to 0.7:1 result in ultralarge complexes (ULCs) [91,97] with net neutral surface charge [63] and arrays of closely approximated PF4 tetramers [99]. It is believed that these unique, near equimolar PHR complexes cause conformational changes within and/or among PF4 tetramers [99-101], that expose neoepitopes which represent the HIT antibody binding site.

Experimental and clinical studies find a distinction between antigenicity and immunogenicity, that is, between antibody binding or cross-reactivity, and antibody formation or seroconversion. Experiments employing a mouse model to investigate HIT immunogenesis have demonstrated that mouse PF4 (mPF4)/heparin complexes, but not mPF4 alone cause development of mPF4/heparin-reactive antibodies. The higher the concentration of mPF4/heparin complexes the greater was the antibody formation [96]. A somewhat surprising study showed that while the equimolar, charge neutral mPF4/heparin ratios resulted in the largest and most antigenic complexes, smaller, high PHR complexes (i.e., PF4>>heparin) with net positive surface charge caused greater mPF4/heparin antibody formation [63]. Various clinical studies conclude that the risk of HIT seroconversion is far less with low molecular weight heparins (LMWHs) compared to unfractionated heparin, yet in in vitro assays, LMWHs cross-react with HIT-antibodies to cause maximal platelet activation [102-104]. Cases of HIT antibody seroconversion have been reported in patients treated with the pentasaccharide, fondaparinux [105]. Surprisingly, the fondaparinux-elicited antibodies cross-react with heparin and LMHWs, but not fondaparinux, in vitro [106,107]. Thus PF4/heparin complexes that bind the most HIT antibodies might not be identical to those which provoke *de novo* antibody generation [108].

It is also interesting to consider that anticoagulant ratios of PF4 and heparin differ from those of antigenic or immunogenic complexes. Only heparin in excess of PF4 has anticoagulant effect [109]. Heparin is neutralized by PF4 present in vitro in PHRs down to as low as 0.42:1 (i.e., PF4<heparin). Heparin would be neutralized by PF4/heparin complexes in the equimolar range associated with maximal HIT antibody binding [110-112]. It is difficult to attribute the process of immunization to the PF4/heparin ratios that would be present during effective anticoagulation. On the contrary, the possibility that higher PHRs may be more immunogenic would explain why minimal heparin exposure, such as heparin flushes [113,114], and lower relative dosage, such as prophylactic vs therapeutic heparin [64,91] are often highly immunogenic. While much has been learned about the physiochemical characteristics of PF4/heparin complexes in which HIT antibody binding sites are exposed, the nature of the in vivo immunogen is less well understood.

### PF4 and innate immunity

With the discovery that a specific PF4/heparin complex was the HIT antigen [13,115] it seemed that the “foreignness” of the heparin-bound PF4 conformation elicited the immune response and the generation of antibodies. Yet studies using antibodies isolated from HIT patients soon demonstrated that PF4 bound to other glycosaminoglycan drugs could also be targeted by PF4/heparin antibodies [116]. These antibodies also bind to PF4 on endothelial cells [13], monocytes [77] or platelets [78,117], or to PF4 immobilized on anionic surfaces [94]. While the conformational neoepitope can be exposed by other PF4 binding partners, none are as immunogenic as unfractionated heparin. That is, they were far less likely to elicit *de novo* antibody formation, suggesting that the impetus for the HIT immune response may be more complex than the presence of a conformational change in a self-protein.

The HIT immune response has several unique aspects, and is as yet, not completely understood [118]. Adaptive, or acquired, immune responses are characterized by antigen-specific antibodies of the IgG isotype, and by immune memory for efficient response to subsequent antigen exposure. Acquired responses are relatively slow to occur, as antibody producing B-cells work with T-cells which recognize specifically presented epitopes of the target. A more immediate, less specific B-cell response occurs in response to generic classes of pathogenic organisms and is independent of past exposure. This rapid innate response is characterized by a less specific, more transient population of IgM antibodies [119]. The HIT immune response is unique. It is characterized by PF4/GAG specific antibodies that occur after only several days of heparin exposure. Despite the rapid appearance, HIT antibodies are often of the IgG isotype. Yet, HIT antibody titers decrease rapidly and there is no memory B-cell response. HIT immunogenesis is not typical of either the innate or the adaptive response, but shares characteristics of each [120,121].

In addition to their role in hemostasis, platelets are increasingly recognized as immune effector cells [122,123]. PF4 is a member of a highly conserved family of host defense effector polypeptides, kinocidins, which display both antimicrobial and leukocyte chemotactic activity [124,125] and play a role in the actions of both the innate and adaptive immune systems [126]. PF4 and other kinocidins contain a signature cationic, amphipathic motif that interacts with and disrupts charged lipid membranes. In its antimicrobial role, PF4 binds to specific species of bacteria, fungus [124,125] and parasites [127,128] facilitating immune defense responses [125,129].

This innate immune role of PF4 may help explain the unusual immune response to PF4 in the presence of heparin. In its antimicrobial role, PF4 binds to anionic components of bacterial surfaces. It has been discovered that PF4 bound to bacteria can be used to affinity enrich HIT antibodies from patient sera, thus demonstrating that antibodies generated in response to heparin therapy cross react with PF4 epitopes exposed on bacterial cells [120]. There is also accumulating evidence that the converse is true, that antibodies occurring naturally in response to microbial infections recognize PF4/heparin complexes. PF4/heparin-reactive IgG and IgM antibodies have been detected in up to 6% of the normal population [120,130,131]. Otherwise healthy individuals with a bacterial periodontal infection, but not exposed to heparin, have measurable PF4/heparin-cross reactive antibodies in proportion to the severity of their disease [132]. And “spontaneous HIT” has been described in patients who developed clinical symptoms and HIT-reactive antibodies without history of heparin exposure, especially in cases of recent bacterial infection [133]. Thus an immune response to endogenous, PF4-bound microbial targets could explain the occurrence of PF4/heparin cross-reactive antibodies in heparin naïve patients or the common observations that seriously ill or septic patients are at greater risk of developing HIT in the presence of heparin [134-136]. These studies suggest a similarity between PF4 bound to microbes and PF4 bound to heparin or to vascular cells.

A direct test of the concept that endogenous PF4-bound target antigens resemble the antigen generated by heparin during anticoagulant therapy used a mouse model of polymicrobial bacteria sepsis, and demonstrated that bacterial exposure resulted in development of PF4/heparin-reactive antibodies with a time course of a typical primary immune response [120]. These studies support the concept that HIT antibodies may resemble naturally occurring antibodies elicited by PF4 functioning as an antimicrobial agent [120,137]. This provides a context to understand how anticoagulant therapy may provoke antibody formation, as the presence of PF4 in complexes with heparin or expressed on the surface of vascular cells may mimic the presentation of PF4 bound to a pathogen, triggering a protective, innate immune response.

### Heparin as an immune adjuvant

Naturally occurring soluble proteins are poorly immunogenic in the absence of an adjuvant such as alum or various oil emulsions, which have been used empirically as immunostimulatory agents [138]. Adjuvants organize surface antigenic epitopes; proteins expressed in a repetitive and ordered fashion are much more immunogenic than in soluble form, and may directly crosslink B-cell receptors (BCRs) [139]. Heparin displays PF4 in closely spaced, repetitive, ridge-like arrays creating polymeric repeating epitopes [99]. In this regards, heparin may serve as an adjuvant that results in an innate-immune response to PF4.

Cells of the immune system express a variety of pattern recognition receptors (PRRs), including toll-like receptors (TLRs). These receptors respond to pathogen associated molecular patterns (PAMPs) that are characteristic of pathogen groups, but distinct from “self”, allowing a limited number of receptors to recognize a great variety of pathogens [140]. Pattern recognition receptors are “threat detectors” that initiate signals to other immune cells [141]. It is becoming clear that commonly used adjuvants activate PRRs and that innate immune responses are central to their effectiveness [142,143]. Indeed, recently there is a focus on discovering novel ligands of PRRs for use as adjuvants to increase efficiency of vaccine development [138]. It is possible that specific PF4/heparin complexes display the antimicrobial conformation of PF4 as a pathogenic molecular pattern and activate these receptors. Heparin also increases the immunogenicity of cationic binding partners, such as IL-8, neutrophil activating peptide-2 and protamine sulfate [144-146]. Mouse immunization experiments have demonstrated that heparin increases immunogenicity of the cationic proteins, protamine and lysozyme, and that the immune responses resemble PF4/heparin seroconversion [145]. Thus, one hypothesis is that heparin functions as an adjuvant, by creating peptide motifs that act as agonists for innate immune pattern recognition receptors.

TLR activation is central to both innate and adaptive immune responses. Specific TLRs respond to particular pathogen classes, generating a context-specific, unique profile of cytokine signals which modulate the magnitude and fine structure of the B cell antibody response [142]. In this way, innate immune recognition of PAMPs provides information on the nature of a pathogen in order to activate and orchestrate the most effective adaptive effector response [138]. Prolonged receptor engagement is required for lymphocytes and dendritic cell differentiation and proliferation to result in plasma cells with high affinity IgG and memory B cells [142]. In contrast, to quickly neutralize replicating pathogens, a more rapid response can be evoked by TLR agonist-mediated activation of dendritic cells and specific B cell subsets to produce IgM as well as class-switched IgG and IgA through a T cell-independent pathway [119,147]. Co-stimulation of TLRs and BCRs can initiate rapid antimicrobial antibody responses to contain pathogen loads until the T-cell dependent antibody responses peak [142]. The balance between the innate and adaptive immune response could depend on the concentration and duration of antigen exposure. In the case of HIT, a persistent high level of the PF4 target antigen might support an adaptive immune response, whereas a more transient exposure might result in only T-cell independent antibody production with the absence of an immune memory response. There is evidence of both types of immune response in HIT [120]. The hypothesis that complexes of PF4 with heparin resemble a conserved pathogenic molecular pattern closely enough to activate TLRs may help explain aspects of the immunogenesis of HIT.

### Prevention/treatment strategies

To date, strategies to prevent or treat HIT have focused on minimizing the use of unfractionated heparin in favor of LMWHs or direct thrombin inhibitors [148]. These alternative anticoagulants have important drawbacks; they are more expensive and complex to manage than heparin and pose risk of bleeding complicated by the absence of effective reversal agents [8]. Focusing on the central role of PF4 in the pathogenesis of HIT allows us to appreciate novel approaches to prevent or treat this syndrome.

As discussed above, HIT antibodies are necessary but not sufficient to cause the intense platelet activation that leads to thrombocytopenia and/or thrombosis. Formation of platelet-activating immune complexes depends on availability of the PF4 target antigen, and the risk of HIT is therefore highest in settings characterized by intense PF4 release. It is logical that minimizing the availability of PF4 or otherwise preventing formation of PF4/heparin complexes would be strategies to abrogate the risk of immunogenesis and pathogenesis of HIT antibodies [149].

One such strategy was suggested by observation of patients with familial hypercholesterolemia. These patients do not achieve adequate low-density lipoprotein (LDL) cholesterol reduction through diet or statin therapy and may undergo frequent LDL apheresis treatments. Despite the repeated exposure to heparin and predisposition to vascular disease, the incidence of HIT is low in this population [150]. Based on this observation, investigators studied the level of PF4 in plasma and on the surface of platelets before and after apheresis. Both plasma and surface PF4 were significantly reduced by the procedure. This may explain the lack of immunogenesis in spite of frequent heparin exposure in these patients. In addition this could prove to be a therapeutic strategy to reduce antigen availability in seropositive patients at high risk for HIT [150].

Presentation of the PF4 target antigen results from the physiochemical properties of complexes of heparin and PF4 tetramers formed and sustained at specific molar ratios [97]. In these highly ordered complexes heparin binding allows close approximation of specific amino acids on PF4 tetramers that create the antigenic epitope [99]. Two recent studies demonstrate that disrupting the tetrameric organization of PF4 by amino acid substitutions [151] or by small inhibitor molecules targeted to the dimer-dimer interface [152] prevents formation of ULCs. Complexes of variant PF4 and heparin were poorly recognized by HIT antibodies [151], and PF4 antagonist molecules inhibited HIT antibody-mediated platelet activation [152]. These studies demonstrate that strategies to alter or diminish the PF4 target antigen may lead to novel therapeutic approaches for treatment of HIT [149,153].

In general, antigenic epitopes are exposed when PF4 binds to any heparin-derived anticoagulant drug. In addition to its anticoagulant activity heparin has potent anti-inflammatory properties; however, the risk of bleeding prevents its use for non-thrombotic indications. Heparin which is desulfated at the 2-O and 3-O positions (ODSH) retains anti-inflammatory properties but has reduced anticoagulant activity [154]. ODSH retains the ability to bind and form complexes with PF4, however, it does not cause platelet activation in the presence of HIT antibodies, suggesting that it does not expose antigenic PF4 target [155]. ODSH can compete with immobilized heparin for PF4 binding and can displace PF4 from cell surfaces [156,157]. When combined with heparin, ODSH reduces immunogenicity in vivo [157] and ameliorates HIT antibody mediated platelet activation in vitro [155,156]. When used together, the capacity of ODSH to sequester a proportion of available PF4 without generating immunogenic complexes may be an effective way to shift the PF4/heparin ratio toward less antigenic complexes. In addition, the availability of less PF4 to block AT binding and cause heparin neutralization could potentially increase anticoagulant potency [158]. Thus the anti-inflammatory, non-anticoagulant properties of ODSH may be useful for increasing the safety and effectiveness of other anticoagulants [159]. It is a particular advantage that ODSH has already undergone trials demonstrating that it can be safely administered to humans [160].

### Conclusions

A determining factor in the risk that HIT antibodies will be elicited as a result of heparin anticoagulant therapy is the presence of PF4. The presence of PF4 also determines whether HIT antibodies will lead to thrombocytopenia and/or thrombosis because only immune complexes of antibody plus target antigen, not antibodies alone, mediate the pathogenic platelet activation. This review presents the hypothesis that heparin serves as an adjuvant, which facilitates antibody formation by displaying PF4 in a motif recognized as a pathogen associated molecular pattern, an agonist for pattern recognition receptors on immune cells. Techniques aimed toward sequestering PF4 or minimizing its conformational alteration are promising areas of research toward developing effective clinical interventions to prevent or treat HIT.

## Abbreviations

PF4: Platelet factor 4; PF4/H: Platelet factor 4/heparin; HIT: Heparin-induced thrombocytopenia; GAG: Glycosaminoglycan; ULC: Ultralarge complex; PAMP: Pathogen associated molecular pattern; PRR: Pattern recognition receptor; TLR: Toll like receptor; BCR: B-cell receptor: AT, antithrombin; PHR: PF4 to heparin ratio.

## Competing interests

The authors have no financial or non-financial competing interests to declare.

## Authors’ contributions

MMP and JMW contributed to the conception, drafting and critical review of the manuscript, and both authors have given their final approval.
